# (Ba,Ca)(Ti,Sn)O_3_-based piezoelectric ceramics promotes neuroprotection by regulating microglial IL-6/JAK2/STAT3 signaling pathway

**DOI:** 10.3389/fncel.2026.1793008

**Published:** 2026-03-25

**Authors:** Haiwang Song, Geng Tang, Yumei Li, Baofei Sun, Zijiang Yu, Mudan Zhang, Dan Yang

**Affiliations:** 1 Department of Human Anatomy, School of Basic Medicine, Guizhou Medical University, Guiyang, Guizhou, China; 2 Key Laboratory of Human Brain Bank for Functions and Diseases of Department of Education of Guizhou Province, College of Basic Medical, Guizhou Medical University, Guiyang, Guizhou, China; 3 College of Mechanical and Electrical Engineering, Guizhou Normal University, Guiyang, Guizhou, China; 4 Department of Radiology, Guizhou Provincial People’s Hospital, Guiyang, Guizhou, China

**Keywords:** (Ba,Ca)(Ti,Sn)O_3_, apoptosis, IL-6/JAK2/STAT3 signaling pathway, inflammation, M1/M2 phenotypes, microglia, neuroprotection, oxidative stress

## Abstract

**Objective:**

To investigate whether (Ba,Ca)(Ti,Sn)O_3_-based piezoelectric ceramics (BCTS) provide neuroprotection by inhibiting the IL-6/JAK2/STAT3 signaling pathway in microglia.

**Methods:**

BCTS surface morphology and elemental distribution were analyzed using Scanning Electron Microscopy (SEM) and Energy Dispersive X-ray Spectroscopy (EDX), phase composition was determined via X-ray diffraction (XRD), and hydrophilicity was measured through contact angle analysis. Immunofluorescence (IF), Western blot, and ELISA were employed to evaluate the expression of microglial markers and inflammatory factors in the BV2 injury model and in spinal cord injury rats. Behavioral tests were conducted to evaluate motor function recovery in spinal cord injury rats. PC12 cells were cultured with BCTS-CM (supernatant from BCTS-treated BV2 cells) to assess the IL-6/JAK2/STAT3 signaling pathway expression and its effects on LDH release, antioxidant enzyme activity, apoptotic proteins, and β-III-tubulin expression.

**Results:**

BCTS exhibited a pure perovskite phase, densely packed grains, and favorable hydrophilicity. It did not affect BV2 cell viability but inhibited LPS-induced M1 microglial activation, reducing the expression of TNF-α, IL-1β, and IL-6. Simultaneously, BCTS promoted M2 microglial polarization, upregulating IL-4, IL-10, and TGF-β1. In PC12 cells, BCTS-CM increased cell survival, antioxidant activities, Bcl-2, and β-III-tubulin expression, while decreasing LDH release, MDA content, BAX and Cleaved Caspase-3 expression. BCTS-induced neuroprotection is mediated by the suppression of the IL-6/JAK2/STAT3 signaling pathway, as evidenced by the fact that IL-6 supplementation counteracts this protection while AG490 treatment further reinforces it compared to BCTS-CM alone. In the spinal cord injury rat model, BCTS inhibited the expression of microglia and inflammatory factors at the injury site, while improving the BBB score and reducing the error rate in the grid walking test.

**Conclusion:**

(Ba,Ca)(Ti,Sn)O_3_-based piezoelectric ceramics exhibit neuroprotective effects by inhibiting IL-6 secretion from microglia, thereby preventing the activation of the IL-6/JAK2/STAT3 signaling pathway in neurons.

## Graphical Abstract

**Figure:**
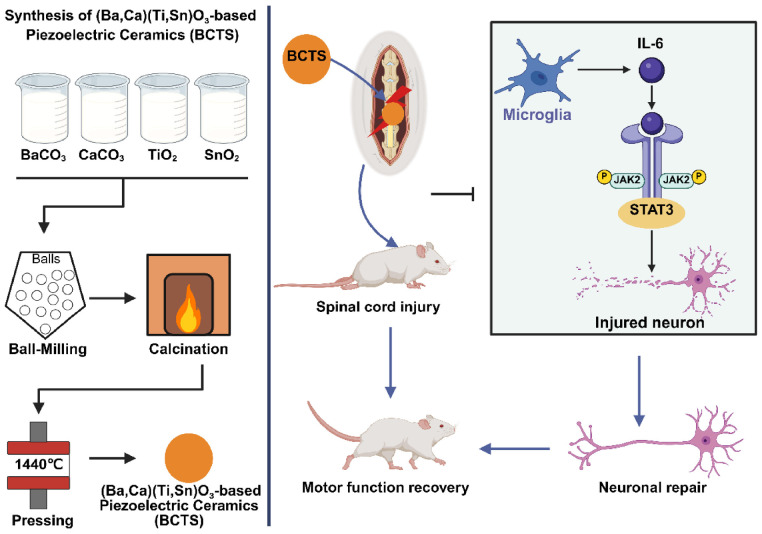
(Ba,Ca)(Ti,Sn)O_3_-based piezoelectric ceramics (BCTS) were fabricated via a solid-state reaction: BaCO_3_, CaCO_3_, TiO_2_, and SnO_2_ powders were mixed, ball-milled, calcined, pressed into discs, and sintered to obtain dense ceramics. The BCTS ceramics attenuate neuronal injury and exert neuroprotective effects by reducing IL-6 release from microglia, thereby suppressing the excessive activation of the IL-6/JAK2/STAT3 signaling pathway in neurons.

## 1 Introduction

Spinal cord injury (SCI) is a common yet devastating condition of the Central Nervous System (CNS), often caused by trauma, compression, or ischemia. It directly impairs sensory, motor, and autonomic functions, frequently resulting in irreversible neurological deficits (Li et al., 2022; Sunshine et al., 2023). These impairments significantly diminish patients’ quality of life and life expectancy, while also imposing substantial physical, psychological, and economic burdens on individuals, families, and society at large (Karamian et al., 2022; Ma et al., 2023). While immediate stabilization is standard, effective neuroprotective strategies that arrest secondary injury mechanisms and promote functional recovery remain a critical unmet clinical need.

Electrical stimulation techniques are well-recognized for their reparative effects on CNS injuries, as they enhance the CNS plasticity (Kathe et al., 2022; Ziesel et al., 2023). However, traditional electrical stimulation techniques are limited by the need for external power sources and wired connections. To overcome these limitations, piezoelectric materials offer a compelling solution by converting physiological mechanical forces—such as respiration and spinal movement—into therapeutic bioelectric signals, enabling self-powered, wireless neuromodulation (Xu et al., 2023; Yan et al., 2022).

Furthermore, the excellent biocompatibility of piezoelectric materials minimizes tissue irritation and immune rejection, making them highly suitable for biomedical applications (Fu et al., 2023). Among lead-free piezoelectric ceramics, (Ba,Ca)(Ti,Sn)O_3_-based piezoelectric ceramics (BCTS) offer an optimal balance of electromechanical performance and physiological stability. While polymers like polyvinylidene fluoride (PVDF) and traditional piezoceramics like ZnO are biocompatible, their relatively low piezoelectric coefficients limit the generation of sufficient electrical output (Kapat et al., 2020). Conversely, high-performance perovskites like (K,Na)NbO3 (KNN) and (Bi,Na)TiO3 (BNT) face challenges *in vivo*: KNN is prone to hydrolytic degradation and ionic leakage in aqueous physiological environments, while BNT raises safety concerns due to potential cytotoxicity and leakage currents (Dong et al., 2022; Xu et al., 2023). Compared to pure BaTiO3, BCTS engineered at the morphotropic phase boundary exhibits significantly enhanced piezoelectric responsiveness, ensuring higher voltage output from mechanical inputs (Liu and Ren, 2009). Consequently, given its superior phase stability, higher piezoelectric constants, and enhanced chemical stability and biocompatibility under physiological conditions (Tang et al., 2025), specifically prepared BCTS were selected to investigate their potential neuroprotective effects.

Despite increasing interest in piezoelectric neuromodulation, its immunomodulatory mechanisms in SCI remain poorly defined. In particular, whether piezoelectric-generated bioelectricity influences microglial polarization and subsequently modulates neuron-specific inflammatory signaling pathways has not been determined. The therapeutic efficacy of BCTS-driven stimulation is likely mediated by the modulation of the immune microenvironment. Microglia, the primary immune cells of the CNS and the most abundant type of glial cells in the nervous system, play a pivotal role in CNS injuries (Borst et al., 2021). They are essential for maintaining neural tissue homeostasis, regulating neuronal activity, and contributing to neural repair processes (Rao et al., 2021; Au and Ma, 2022). Research indicates that microglia exhibit distinct functional roles depending on their phenotypic state (Tao et al., 2021). According to the widely accepted binary classification, M1-type microglia release pro-inflammatory cytokines, including tumor necrosis factor-α (TNF-α), interleukin-1β (IL-1β), and IL-6 (Wang G. et al., 2022; Xian et al., 2022; Liu et al., 2024). These cytokines initiate and exacerbate neuroinflammatory responses, leading to further neuronal and tissue damage (Hui et al., 2023; Yu et al., 2023). Conversely, M2-type microglia secrete neurotrophic factors and anti-inflammatory cytokines such as transforming growth factor-β1 (TGF-β1), interleukin-4 (IL-4) and interleukin-10 (IL-10) (Wang L. et al., 2024; Zhong et al., 2024; Zhu et al., 2024), which inhibit inflammation and promote neuronal regeneration and synapse formation, thereby mitigating neural damage and facilitating tissue repair (Xie et al., 2021; Zou et al., 2024). Therefore, shifting the microglial balance from the destructive M1 state to the reparative M2 state represents a primary mechanism by which piezoelectric stimulation could exert neuroprotection.

To define the precise molecular mechanism, we focused on the crosstalk between microglia and neurons. Among the cytokines released by M1 microglia, Interleukin-6 (IL-6) is a critical mediator of neuronal damage. Excessive IL-6 activates the JAK2/STAT3 signaling pathway in neurons, a cascade implicated in excitotoxicity and structural degradation (Liu et al., 2022; Zhao et al., 2022; Lee J. Y. et al., 2023). We hypothesize that BCTS-mediated bioelectric signaling modulates microglial polarization toward the M2 phenotype, thereby suppressing IL-6 release, limiting neuronal JAK2/STAT3 overactivation, and exerting neuroprotective effects.

In this study, we utilized BCTS to validate this hypothesis. Specifically, we investigated how BCTS-driven stimulation modulates microglial polarization to regulate the IL-6/JAK2/STAT3 signaling pathway in neurons. Our objective is to elucidate the precise neuroprotective mechanisms of BCTS, thereby providing a solid theoretical basis and novel therapeutic strategies for the treatment of spinal cord injury.

## 2 Materials and methods

### 2.1 Materials

JSM-5900 Scanning Electron Microscope (Japan), Xpert-PRO X-ray Diffractometer (Netherlands), Water Contact Angle Goniometer JC2000D1 (Shanghai Zhongchen Digital Technology Equipment Co., Ltd., China), Quasi-static *d*_33_ Meter ZJ-3AN (Institute of Acoustics, Chinese Academy of Sciences, China), Ultrasound Stimulator (GV-CSI1.0) (Green Valley BrainTech (ShenZhen) Medical Technology Co., Ltd. (China), BV2, PC12 (Wuhan Pricella Biotechnology Co., Ltd., China), Rabbit Anti-iNOS (Thermo Fisher Scientific, Inc.), Rabbit Anti-Iba-1, Arg-1, NeuN, Goat Anti-Rabbit IgG H&L (Alexa Fluor 488), Goat Anti-Mouse IgG H&L (Alexa Fluor 594) (Abcam, UK), Rabbit Anti-CD86, CD206, β-actin, HRP-conjugated Goat Anti-Rabbit IgG (H + L) (Wuhan Proteintech Biotechnology Co., Ltd., China), Rabbit Anti-IL-6, TNF-α, IL-4, IL-10, TGF-β1 (ImmunoWay, United States), Rabbit Anti-IL-1β, JAK2, P-JAK2, STAT3, P-STAT3, BAX, Bcl-2, Cleaved Caspase-3 (Shenyang Wanlei Biotechnology Co., Ltd., China), BCA Protein Assay Kit, CCK8 Cell Viability Assay Kit, LDH Cytotoxicity Assay Kit, SOD Activity Assay Kit, CAT Activity Assay Kit, GSH-Px Activity Assay Kit, MDA Assay Kit, Dapi (Beijing Solarbio Science & Technology Co., Ltd., China), LPS (Sigma, Germany), IL-6, AG490 (MCE, Germany), Laser Confocal Microscope (Olympus Corporation, Japan), SDS-PAGE Electrophoresis System (BIO-RAD, USA).

### 2.2 The preparation of BCTS piezoelectric ceramics

The Ba_0.94_Ca_0.06_Ti_0.92_Sn_0.08_O_3_ (abbreviated as BCTS) piezoelectric ceramics were prepared using the traditional solid-state reaction method. The raw materials included barium carbonate (BaCO_3_, 99.8%), calcium carbonate (CaCO_3_, 99.5%), titanium dioxide (TiO_2_, 99.0%), and tin oxide (SnO_2_, 99.99%). After drying, these materials were weighed according to their stoichiometric ratios and mixed using ZrO_2_ balls and anhydrous ethanol as the milling medium for 12 h to ensure thorough blending of the powders. The mixed powders were then dried and sieved, followed by calcination at 1,200°C for 2 h to promote solid-state reactions. The calcined powders were subjected to an additional 12-h milling, drying, and sieving process. The resulting powders were mixed with paraffin, and then pressed into discs with a diameter of 12 mm and a thickness of 1.2 mm under 10 MPa pressure. These discs were sintered in air at 1,440°C for 2 h, with a hold at 600°C for 2 h to complete the removal of paraffin.

### 2.3 Characterization

The surface microstructure of the sintered ceramic samples was observed using a Scanning Electron Microscope (ZEISS GeminiSEM 300, Germany). Since the ceramic samples are insulating materials, they were pre-treated by gold sputtering for 80 s before observation to ensure successful imaging. Additionally, Energy Dispersive X-ray Spectroscopy (EDX) was utilized to track the uniformity of the elemental distribution. Phase analysis of the prepared ceramics was conducted using an Xpert-PRO X-ray Diffractometer under the following conditions: Cu-Kα target (λ = 1.5406 Å), scan speed of 1°/min, tube voltage of 45 kV, tube current of 40 mA, diffraction angle range of 10°–80°, with a step size of 0.039°. The wettability of the piezoelectric ceramics was measured using a water contact angle goniometer (JC2000D1, Shanghai Zhongchen Digital Technology Equipment Co., Ltd., China). To measure the electrical properties, silver paste was first applied to the top and bottom surfaces of the samples and then sintered at 750°C for 30 min to form the electrodes. For ceramics used in biological experiments, thin copper foils were clamped onto both sides of the ceramic samples to create the electrodes. Subsequently, the samples were polarized in a silicone oil bath at room temperature using a DC electric field of 2.5 kV/mm. After polarization, the samples were left for 24 h to release residual stress and charge, and then their piezoelectric constants were evaluated using a quasi-static *d*_33_ meter (ZJ-3AN, Institute of Acoustics, Chinese Academy of Sciences, China).

The variation of the dielectric constant (ε*_*r*_*) with temperature, from 20 to 150°C, was analyzed at frequencies of 100 Hz, 1 kHz, 10 kHz, 100 kHz, and 1 MHz using an LCR meter (TH2618B, Guangzhou Zhuo Yue Electronic Instrument and Equipment Co., Ltd., China). Ferroelectric hysteresis loops (P-E) were recorded at voltage of 1 kV using a ferroelectric tester (RT66A; Radiant Technologies Inc., Albuquerque, NM). To ensure the reproducibility of these material properties, all characterizations were performed on at least three independent samples from different batches, and representative results are presented.

### 2.4 Experimental animals

Adult Sprague-Dawley rats (equal numbers of males and females, 200–250 g) were obtained from the Experimental Animal Center of Guizhou Medical University (Certificate No.: SCXK(Gui)2025-0001). All experimental procedures were approved by the Ethics Committee of Guizhou Medical University (Approval No.: 2100034) and conducted in accordance with the Guidelines for Ethical Review of Animal Welfare in Experiments (GB/T 35892–2018). Animal handling, surgical procedures, and all experimental manipulations adhered strictly to the Regulations on the Administration of Experimental Animals (China) and the institutional guidelines for laboratory animal welfare and management.

### 2.5 Spinal cord injury model and BCTS intervention

SD rats were anesthetized via intraperitoneal injection with 30 mg/kg of 2% sodium pentobarbital (Ying et al., 2025) and placed in the prone position, after which a midline dorsal incision was made to expose the paraspinal muscles and vertebrae, T9 laminectomy was performed with careful removal of the lateral edges of the T9 articular processes, and the exposed spinal cord was subsequently compressed with an aneurysm clip applying a closing force of 30 g for 10 s (Lee P. H. et al., 2023; Vahabi and Öztürk, 2023). Successful spinal cord injury was confirmed by narrowing of the dorsal vein, hindlimb twisting or twitching, tail curling, and loss of voluntary movement below the injury site, after which hemostasis was achieved with a gelatin sponge and the wound was rinsed with sterile saline. In the intervention group, sterilized BCTS material was applied to the injury site and secured with a small amount of sterile fibrin glue, and the muscles, fascia, and skin were then sutured in layers with the incision disinfected using povidone-iodine. Sham-operated animals underwent the same procedure without spinal cord compression. Postoperatively, rats were individually housed with free access to food and water. Manual bladder expression was performed twice daily until spontaneous urination resumed, and the perineal area was kept dry. Intramuscular penicillin (0.1 mL/day) was administered for three consecutive days to prevent infection.

SD rats were randomly assigned to each experimental group using a computer-generated randomization table to ensure equal numbers of rats in each group: (1) Sham group, in which only laminectomy was performed without inducing spinal cord injury. (2) SCI group, in which spinal cord injury was induced without any further intervention. (3) BCTS group, in which rats received BCTS treatment after spinal cord injury. To activate the piezoelectric effect of BCTS materials, BCTS group rats were anesthetized and placed in the prone position for ultrasound (US) treatment. Each rat received ultrasound stimulation for 15 min per day for 3 consecutive weeks. US stimulation was applied to BCTS using the following parameters: frequency 1 MHz, amplitude 37% of the maximum output, pulse width 500 μs, pulse repetition period 7 ms, stimulation duration 1 s, and burst interval 5 s, corresponding to an acoustic pressure of 1.224 MPa (Zhang et al., 2023).

### 2.6 Cell culture

In this study, we utilized the BV2 cell line as a substitute for microglia and the rat pheochromocytoma PC12 cell line as a substitute for neurons. The BCTS samples were sterilized by soaking in 75% alcohol for 60 min, followed by three 5 min PBS washes and 30 min of UV sterilization. The BV2 cell line was seeded onto the negative polarization side of BCTS at a density of 1 × 10^5^ cells per well, with 3 ml of complete DMEM medium (10% FBS + 1% penicillin/streptomycin) per well, cultured in a 37°C, 5% CO2 incubator with medium changes every 2 days, and passaged upon reaching 80% confluence. In this experiment, 1 μg/ml (Ismail et al., 2020) of LPS was utilized to establish damage models for both the BV2 cell line and the PC12 cell line. The supernatant from BCTS-treated BV2 cells, termed BCTS cell-conditioned medium (BCTS-CM) (Jiang et al., 2023), was collected, centrifuged (5 min at 1,000 × g), and then used to culture PC12 cells (Guo et al., 2016; Guo et al., 2024). Additionally, to verify the effect of the IL-6/JAK2/STAT3 signaling pathway on PC12 cells, the PC12 were treated with IL-6 at a concentration of 10 ng/mL (Wang Y. et al., 2024) and AG490 at 5 μM (Yang B. et al., 2020) in the BCTS-CM. In the BCTS group, ultrasound stimulation was applied with a frequency of 19.876 MHz, amplitude of 100 mVpp, and burst period of 100 ms, with a measured acoustic pressure of 2.45 MPa. BV2 cells were exposed to ultrasound stimulation for 5 min per day for 3 consecutive days (Zhang et al., 2023).

In BV2 cells, they are divided into groups, ① Con group (LPS^–^ and BCTS^–^), ② LPS group (LPS^+^ and BCTS^–^), ③ BCTS group (LPS^+^ and BCTS ^+^). In PC12 cells, they are divided into groups, ① Con group (LPS^–^ and BCTS-CM^–^ and IL-6^–^ and AG490^–^), ② LPS group (LPS^+^ and BCTS-CM^–^ and IL-6^–^ and AG490^–^), ③ BCTS-CM group (LPS^+^ and BCTS-CM^+^ and IL-6^–^ and AG490^–^), ④ IL-6 group (LPS^+^ and BCTS-CM^+^ and IL-6^+^ and AG490^–^), ⑤AG490 group (LPS^+^ and BCTS-CM^+^ and IL-6^–^ and AG490^+^).

### 2.7 Cell viability assay

Cell viability was assessed using the CCK8 assay kit following the manufacturer’s instructions. Cells were plated at 100 μL per well in a 96-well plate and pre-incubated for 24 h. Following this, 10 μL of CCK8 reagent was added to each well, and the plate was further incubated for 2 h before measuring the optical density (OD) at 450 nm using a microplate reader to assess cell viability. Cell viability was calculated according to the following formula: Cell viability (%) = (OD_*treatment*_ – OD_*blank*_)/(OD_*control*_−OD_*blank*_) × 100%. The control cells were considered to have 100% viability.

### 2.8 Analysis of LDH release, antioxidant enzyme activity and MDA levels

To assess lactate dehydrogenase (LDH) levels in the culture medium, supernatants were collected and analyzed using an LDH assay kit according to the manufacturer’s instructions, with cell suspensions undergoing 25 s of ultrasonic treatment followed by centrifugation (4°C, 15 min at 8,000 × g), and the procedures and calculations were based on the manufacturer’s manual. The supernatants were collected, and assay kits were used to measure the activities of superoxide dismutase (SOD), catalase (CAT), glutathione peroxidase (GSH-Px), and the content of malondialdehyde (MDA).

### 2.9 Western blot analysis

At 21 days post-spinal cord injury, rats were anesthetized with 1% sodium pentobarbital, and a 0.5 cm spinal cord segment adjacent to the injury site was dissected. The tissue was homogenized and lysed in protein lysis buffer containing protease inhibitors (PMSF: 100:1, v/v). The mixture was vortexed and centrifuged at 12,000 × g for 15 min at 4°C, and the supernatant was collected for protein quantification. After treatment, BV2 and PC12 cells were washed with 1 × PBS, lysed on ice with RIPA buffer (containing protease and phosphatase inhibitors), and centrifuged at 12,000 × g for 10 min at 4°C. The total protein concentration in each sample was measured using a BCA protein assay kit (Thermo Fisher Scientific, United States). Subsequently, protein samples were mixed with loading buffer and boiled to denature the proteins. Proteins of varying molecular weights were separated by SDS-PAGE using polyacrylamide gels of appropriate concentrations, and then transferred to PVDF membranes. The membranes were blocked with 5% non-fat milk for 90 min to prevent non-specific binding. After blocking, the membranes were incubated overnight at 4°C with primary antibodies specific to the target proteins. The next day, the membranes were incubated with secondary antibodies at room temperature for 120 min, washed three times with 1 × TBST. The specific dilution ratios of the antibodies used in the experiments are detailed in Table 1). Immersed in ECL detection reagent, and protein bands were visualized using a chemiluminescence detection system. β-actin was used as a loading control, and the bands were analyzed using ImageJ software.

**TABLE 1:** Information of primary and secondary antibodies for western blot.

Target protein	Dilution
Arg-1 (WL02825) BAX (bs0127R)	1:2,000 1:1,500
Bcl-2(bs-0038R) CD206 (18704-1-AP) CD86 (13395-1-AP)	1:1,000 1:2,000 1:2,000
Cleaved Caspase-3 (WL01992)	1:1,000
Il-10 (WL03088)	1:2,000
IL-1β (WL02257) IL-4 (WL05916) IL-6 (WL02841) iNOS (WL0992a)	1:1,000 1:3,000 1:3,000 1:1,500
JAK2 (WL02188) P-JAK2(WL02997)	1:1,000 1:1,000
P-STAT3 (WL06214)	1:1,000
STAT3 (WL01836)	1:1,000
TGF-β1(26155-1-AP)	1:3,000
TNF-α (17590-1-AP)	1:1,500
β-actin (20536-1-AP)	1:5,000
HRP-conjugated Goat Anti-Rabbit IgG (SA00001-2)	1:10,000

### 2.10 Immunofluorescence analysis

At 21 days post-spinal cord injury, rats were anesthetized and perfused with PBS followed by 4% paraformaldehyde (PFA) for fixation. The spinal cord segments were then dehydrated using a sucrose gradient and embedded for sectioning. Routine frozen sections were prepared. The sections were washed 3 times with 1 × PBS for 5 min each, followed by fixation with 4% PFA at 4°C for 20 min, and two additional washes with PBS. For cell samples, cells were seeded onto coverslips and allowed to adhere. After sufficient attachment, the coverslips were washed twice with 1 × PBS, followed by fixation with 4% PFA at 4°C for 20 min and two additional PBS washes. Primary antibodies were added and incubated overnight at 4°C, followed by gentle washing with PBS. The coverslips were then incubated with fluorescence-labeled secondary antibodies at room temperature for 120 min, washed with PBS, and stained with DAPI for 20 min. The specific dilution ratios of the antibodies used in the experiments are detailed in Table 2. After rinsing twice with PBS, the coverslips were mounted and observed under a laser confocal microscope. Fluorescence intensity was analyzed using ImageJ software.

**TABLE 2:** Information of primary and secondary antibodies for Immunofluorescence.

Target protein	Dilution
iNOS (MA5-17139)	1:250
Dapi (10 μg/mL) (D3571)	1:500
CD206 (18704-1-AP)	1:100
β-III-tubulin (ab18207)	1:200
Iba-1 (ab153696)	1:200
NeuN (ab177487)	1:250
Dapi (10 μg/mL)	1:500
Goat Anti-Rabbit IgG H&L (Alexa Fluor 488) (ab150077)	1:500
Goat Anti-Mouse IgG H&L (Alexa Fluor 594) (ab150116)	1:500

### 2.11 ELISA analysis

Collect the cell culture supernatant and centrifuge (10 min at 1,000 × g) to remove cell debris, and the levels of inflammatory cytokines (TNF-α, IL-1β, IL-6, TGF-β1, IL-4, and IL-10) were quantified according to the ELISA kit instructions, with absorbance measured at 450 nm using a microplate reader.

### 2.12 Behavioral tests

Basso-Beattie-Bresnahan (BBB) scale: Hindlimb motor function was assessed using the Basso, Beattie, and Bresnahan (BBB) locomotor rating scale at specific time points following spinal cord injury, as well as preoperatively and weekly up to nine weeks post-surgery, excluding intervention days. Evaluations were conducted in a 100 cm × 100 cm open-field arena, where rats were allowed to move freely for 4–5 min. The BBB scale ranges from 0 (no observable hindlimb movement) to 21 (normal locomotor function). Two trained and blinded observers independently scored each rat based on hindlimb joint movement, weight support, coordination, trunk stability, toe clearance, and tail position.

Grid walking test: The grid-walk test was used to assess hindlimb motor function in rats. Prior to the experiment, rats were allowed to explore the grid for 5 min to acclimate to the environment. A 1.5 m × 1.5 m metal grid with squares measuring 5 cm × 5 cm was used for the test. The rats were placed on the grid and allowed to walk freely. The experiment was conducted by two blinded observers: one observer recorded the total number of steps taken by the rats, while the other recorded the number of hindlimb foot drops. Foot drops were defined as instances when the rat’s hind paw dropped below the grid during stepping, failing to maintain stable support. A foot fault was recorded when the hind paw completely fell into a square. The total number of foot faults and the total number of steps were recorded to calculate the average foot fault rate. The above data were obtained by two independent investigators who were blinded to the treatment assignments.

### 2.13 Motor evoked potentials

At 21 days post-spinal cord injury, rats were anesthetized, and recording electrodes were inserted into different regions of the ipsilateral gastrocnemius muscle, with a ground electrode placed subcutaneously at the neck. A stimulating electrode was positioned 5 mm rostral to the injury site to evoke motor-evoked potentials, and both amplitude and latency were recorded. Electrical stimulation was delivered at 200 mV and 1 Hz. MEPs were monitored and recorded for each group using electrophysiological acquisition software.

### 2.14 Statistical analysis

Data analysis was performed using SPSS 22.0, and statistical graphs were generated using GraphPad Prism 8.0, with all data presented as mean ± standard error of the mean (SEM) and assessed for normality and homogeneity of variance before statistical testing. For comparisons among multiple groups, one-way ANOVA followed by Tukey’s *post-hoc* test was used. For comparisons between two groups, Student’s *t*-test was applied. All tests were performed on data that met the assumptions of the corresponding statistical methods.

## 3 Results

### 3.1 BCTS characterization

**FIGURE 1:**
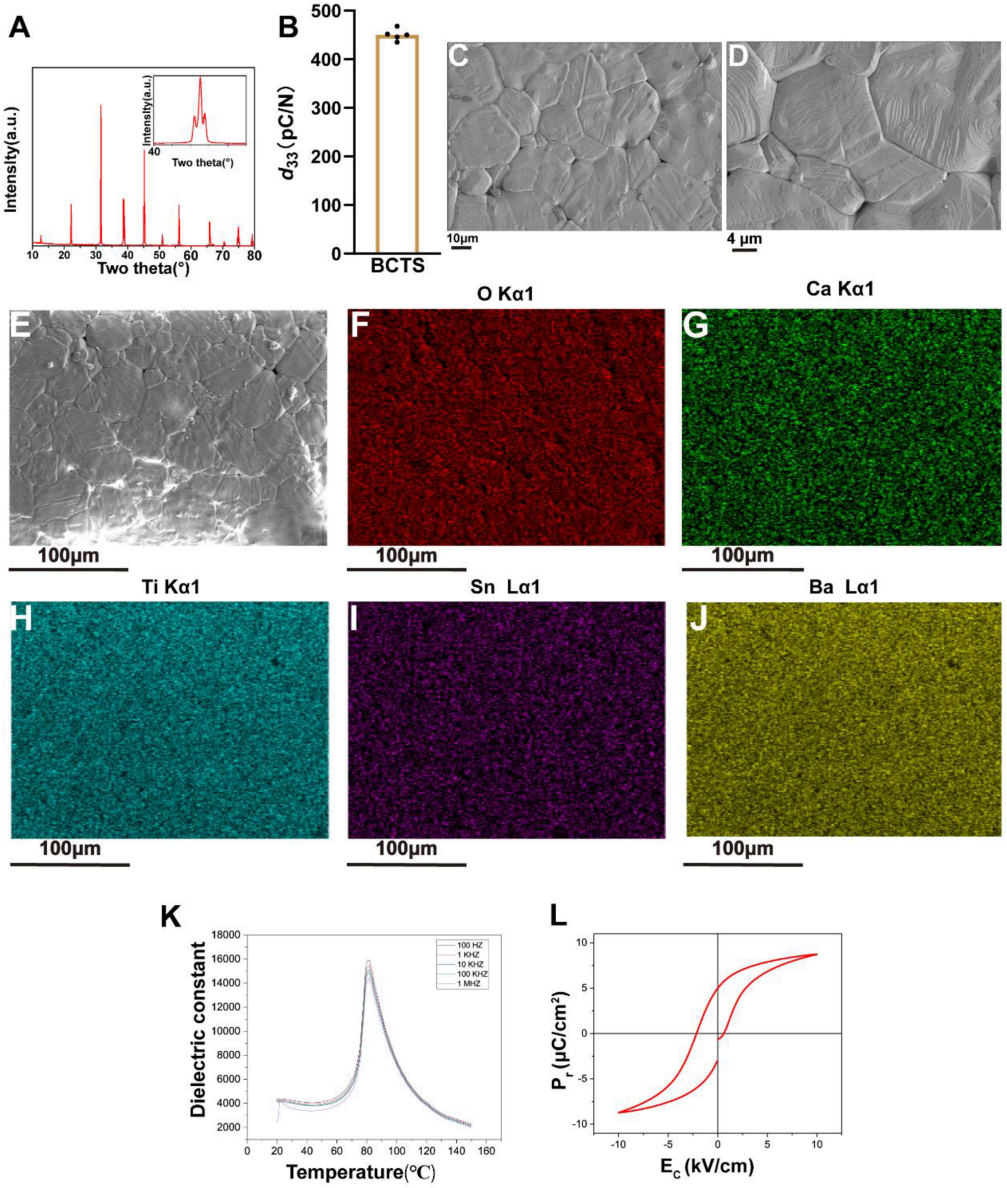
BCTS characterization. **(A)** XRD spectrum of BCTS. **(B)** The *d*_33_ constant of BCTS. **(C,D)** SEM images showing the appearance of BCTS at different magnifications. Scale Bar = 10 and 4 μm. **(E–J)** Elemental mapping EDS spectra for O, Ca, Ti, Sn, and Ba. Scale Bar = 100 μm. **(K)** Temperature-dependent dielectric constant (εr) of BCTS ceramics measured over a temperature range of 20–150°C at frequencies of 100 Hz, 1 kHz, 10 kHz, 100 kHz, and 1 MHz. **(L)** Ferroelectric hysteresis loop of the BCTS ceramics, which exhibits typical characteristics of a relaxor ferroelectric material.

The XRD pattern of BCTS is shown, where the peaks correspond to the BaTiO_3_ perovskite structure, with no additional peaks observed, indicating the absence of any secondary phases in the ceramic. Additionally, the inset in the figure shows a distinct peak splitting near 2θ = 45° (Figure 1A), indicating the characteristic of multiphase coexistence. The presence of multiple phases in piezoelectric ceramics is beneficial for the deflection of dipoles, thereby enhancing piezoelectric properties (Zhu et al., 2013). Furthermore, the measured *d*_33_ value of BCTS is 450 ± 12 pC/N (Figure 1B). The microstructure of BCTS (Figures 1C,D) reveals the tightly packed grain arrangement of the piezoelectric ceramic. Elemental mapping images (Figures 1E–J) demonstrate uniform distribution of O, Ca, Ti, Sn, and Ba, indicating that the doped Ca^2+^ and Sn^4+^ have successfully integrated into the BaTiO_3_ lattice. Figure 1K show that at all measured frequencies, the dielectric constant initially increases with temperature, reaches a maximum value around 81.7°C (corresponding to the Curie temperature, Tc) and then decreases. At 37°C (physiological temperature), the dielectric constant ranges from 3426 to 3897, exhibiting a favorable dielectric response at body temperature. Studies have shown that piezoelectric materials with good dielectric properties can enhance the electrical coupling effect at the cell–material interface and promote the functional expression of electroactive cells (Jacob et al., 2018). Furthermore, the Curie temperature (81.7°C) of this material is significantly higher than physiological temperature, ensuring its structural stability and electrical performance retention under *in vivo* application conditions. Figure 1L show that the remanent polarization (*P*_*r*_) was measured to be 4.99 μC/cm^2^, confirming the presence of switchable polarization and retained charge storage capability in the material. This retained polarization indicates that BCTS piezoelectric ceramics are capable of maintaining surface charge, which may locally modulate the electrical microenvironment at the cell–material interface. Such charge accumulation could potentially influence the activity of membrane receptors on neuronal cells (Thrivikraman et al., 2018), thereby affecting intracellular signaling pathways and subsequent cellular behaviors

### 3.2 BCTS displays excellent biocompatibility

**FIGURE 2:**
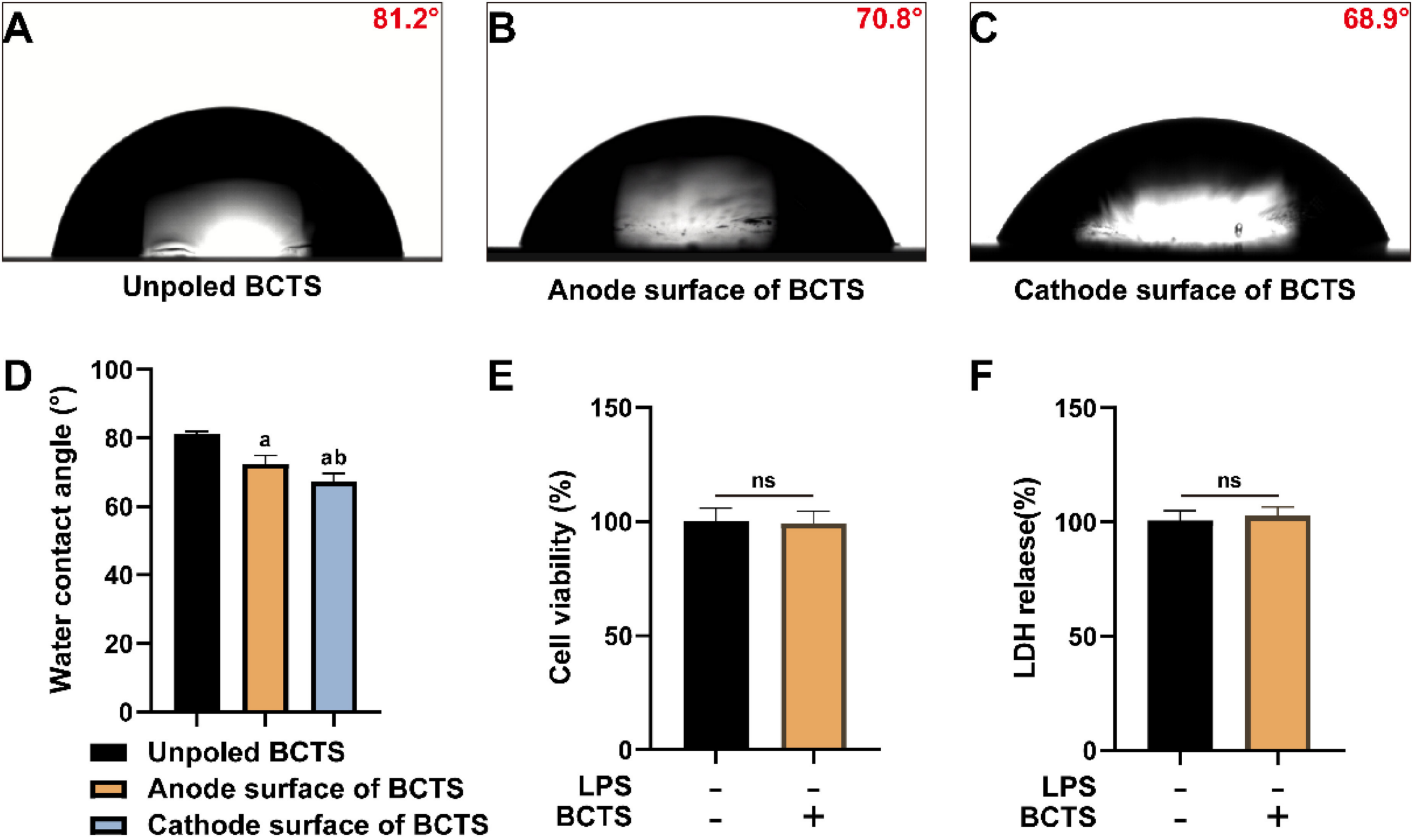
BCTS displays excellent biocompatibility. **(A–D)** Representative water contact angle results for BCTS. Data are expressed as mean ± SEM, *n* = 3. **(E)** Statistical analysis of BV2 cell viability after BCTS intervention. Data are expressed as mean ± SEM, *n* = 5. **(F)** Statistical analysis of LDH release from BV2 cells following BCTS intervention. Data are expressed as mean ± SEM, *n* = 5 vs. Unpoled BCTS group, ^a^*P* < 0.05. vs. Anode surface of BCTS group, ^b^*P*<0.05. ns, no significance.

The hydrophilic property of a material plays a vital role in maintaining cell viability after implantation, as it enhances cell-substrate interactions and promotes cellular metabolism. The wettability results of BCTS (Figures 2A–C) indicate that the material exhibits excellent hydrophilicity. The water contact angle (WCA) of unpoled BCTS was measured to be 81.14 ± 0.8°, while the WCA values for the anode and cathode surfaces of BCTS were 72.42 ± 2.5° and 68.82 ± 2.3°, respectively (Figure 2D). Based on these results, the cathode surfaces of BCTS were selected for subsequent cell seeding. Furthermore, the effects of BCTS on BV2 cell viability were evaluated using CCK8 and LDH assays. The results (Figures 2E,F) demonstrated that BCTS did not exert any adverse effects on BV2 cell viability or LDH release, indicating that BCTS possesses excellent biocompatibility.

### 3.3 BCTS promoted LPS-induced polarization of BV2 microglial cells toward the anti-inflammatory M2 phenotype

**FIGURE 3:**
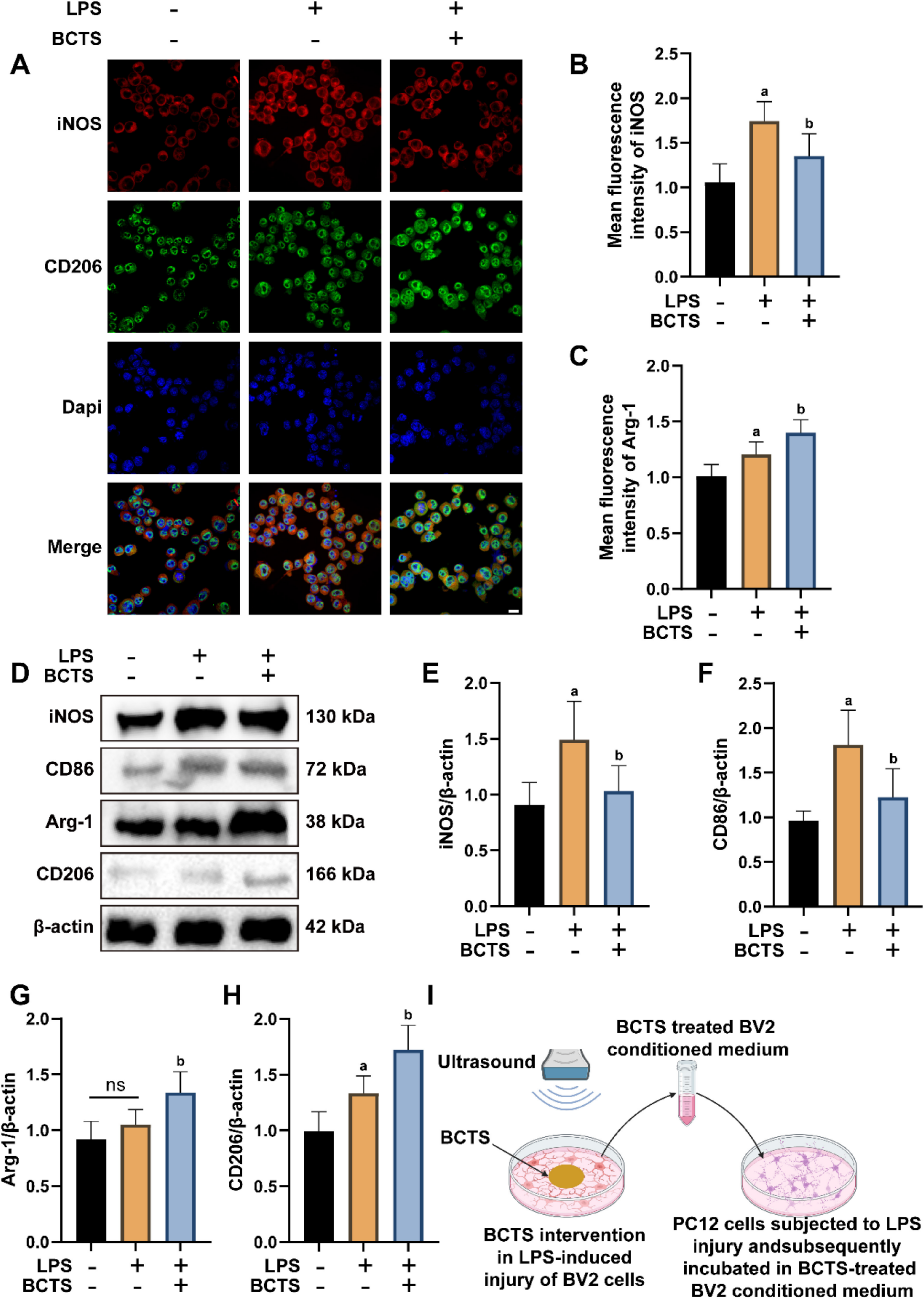
BCTS promoted M2 polarization of LPS-induced BV2 microglia. **(A–C)** the immunofluorescence results and statistical analysis of the M1 marker iNOS and M2 marker CD206 in microglial cells. **(D–F)** Western blot results and statistical analysis of iNOS and CD86 expression. **(G,H)** Western blot results and statistical analysis of Arg-1 and CD206 expression. **(I)** Schematic of the experimental procedure: LPS-injured BV2 cells were treated with ultrasound-driven BCTS, and the resulting conditioned medium was used to culture LPS-injured PC12 cells. Data are expressed as mean ± SEM, *n* = 5. Scale bar = 20 μm vs. Con group, ^a^*P* < 0.05, ns, no significance vs. LPS group, ^b^*P* < 0.05.

To explore the impact of BCTS on the polarization of BV2 microglial cells (Figure 3I), we utilized IF staining, Western Blot to assess the expression of M1 and M2 markers in BV2 cells. IF staining results revealed that LPS treatment significantly increased the iNOS expression in BV2 cells, indicative of M1 polarization. However, BCTS intervention markedly reduced the iNOS expression compared to the LPS group (*P* = 0.0447). In contrast, BCTS intervention markedly increased the Arg-1 expression compared to the LPS group (*P* = 0.0434) (Figures 3A–C). The trend observed in Western Blot is consistent with the IF staining. Specifically, after BCTS intervention, the expression of iNOS (*P* = 0.0425) and CD86 (*P* = 0.0222) (Figures 3D–F) was lower than that in the LPS group, while the expression of Arg-1 (*P* = 0.0371) and CD206 (*P* = 0.0145) was higher than in the LPS group (Figures 3G,H). The schematic model is presented in Figure 3I. Collectively, these findings suggest that BCTS effectively inhibited the pro-inflammatory M1 polarization while promoting the anti-inflammatory M2 polarization of BV2 microglial cells.

### 3.4 BCTS attenuates the inflammatory response in the LPS-induced BV2 injury model

**FIGURE 4:**
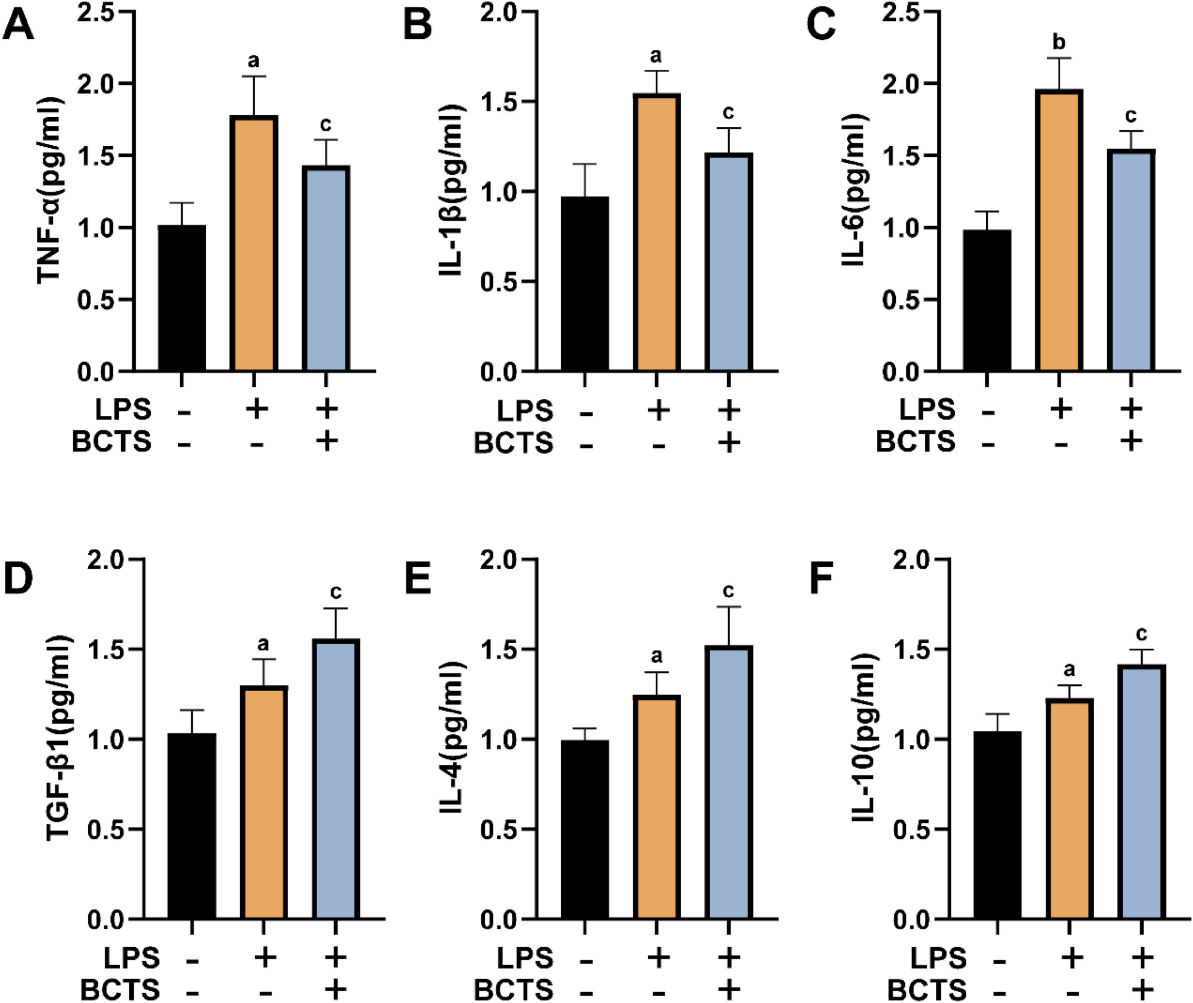
BCTS reduced inflammation in the LPS-induced BV2 injury model. **(A–C)** Statistical analysis of the expression of pro-inflammatory cytokines TNF-α, IL-1β and IL-6. **(D–F)** Statistical analysis of the expression of anti-inflammatory cytokines TGF-β1, IL-4 and IL-10. Data are expressed as mean ± SEM, *n* = 5 vs. Con group,^a^*P* < 0.05, ^b^*P* < 0.01 vs. LPS group, ^c^*P* < 0.05.

To investigate the effect of BCTS on inflammatory factors following microglial polarization, ELISA was performed to measure the levels of pro-inflammatory cytokines TNF-α, IL-1β, and IL-6, as well as anti-inflammatory cytokines TGF-β1, IL-4, and IL-10 in the supernatant of BV2 cells. The results showed that, compared to the LPS group, the concentrations of pro-inflammatory cytokines TNF-α (*P* = 0.0481), IL-1β (*P* = 0.0112), and IL-6 (*P* = 0.038) were reduced in the BCTS group (Figures 4A–C), while the concentrations of anti-inflammatory cytokines TGF-β1 (*P* = 0.0418), IL-4 (*P* = 0.0311), and IL-10 (*P* = 0.0101) were increased (Figures 4D–F). These findings suggest that BCTS suppresses the pro-inflammatory response and enhances the anti-inflammatory response, thereby modulating the inflammatory response following microglial polarization.

### 3.5 BCTS-CM alleviates PC12 cell injury by inhibiting the IL-6/JAK2/STAT3 signaling pathway

**FIGURE 5:**
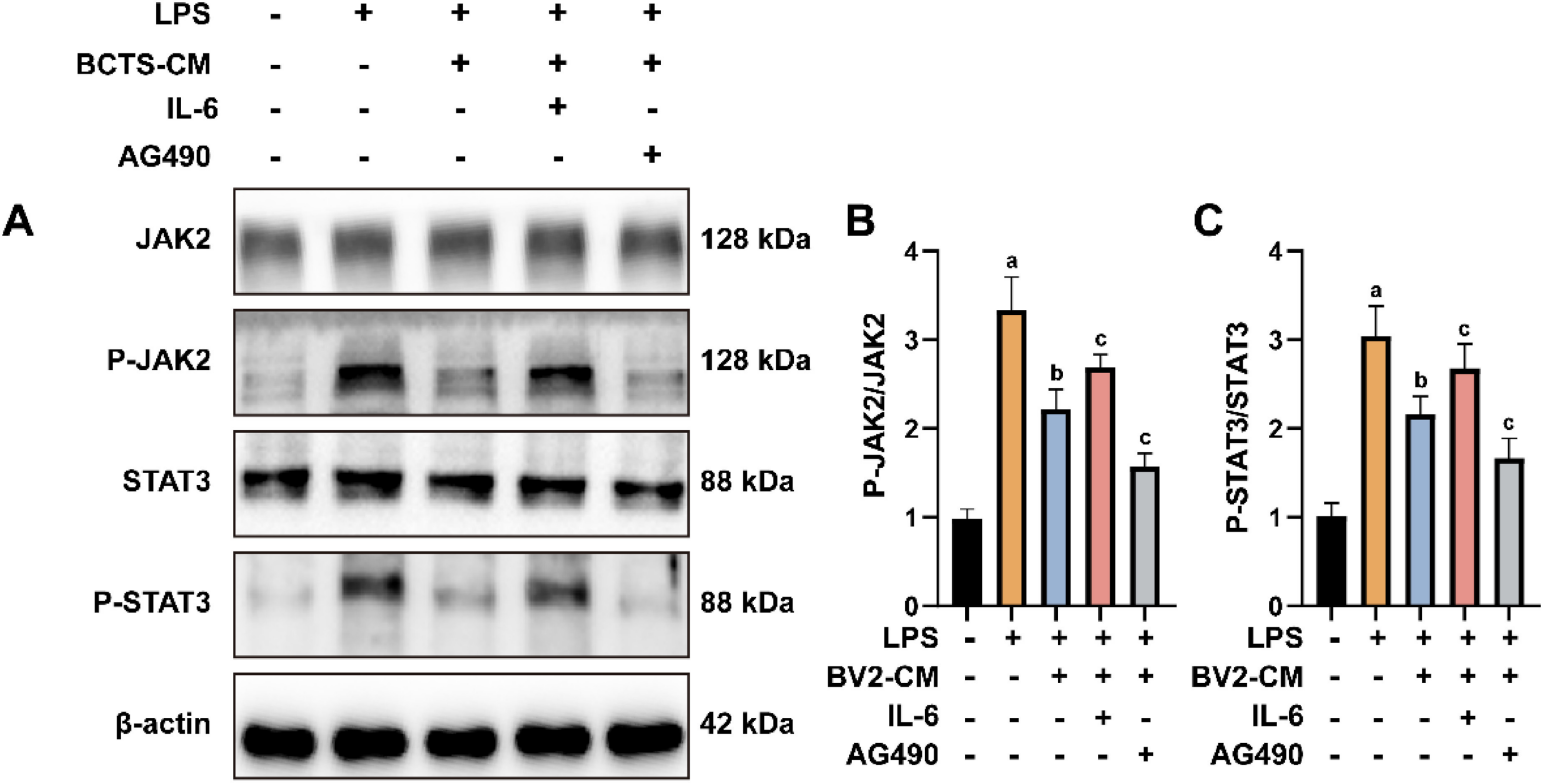
BCTS-CM attenuates activation of the IL-6/JAK2/STAT3 pathway in PC12 cells. **(A)** Western blot results of IL-6/JAK2/STAT3 signaling pathway. **(B,C)** Statistical analysis of P-JAK2/JAK2 and P-STAT3/STAT3 expression. Data are expressed as mean ± SEM, n = 5 vs. Con group, ^a^*P* ≤ 0.01 vs. LPS group, ^b^*P* ≤ 0.05 vs. BCTS-CM group, ^c^*P* ≤ 0.05.

To investigate whether polarized BV2 cells could exert neuroprotective effects by inhibiting the IL-6/JAK2/STAT3 signaling pathway, the conditioned medium from BCTS-treated BV2 cells (BCTS-CM) was collected and used to culture PC12 cells subjected to LPS-induced injury. The expression of proteins related to the IL-6/JAK2/STAT3 signaling pathway were analyzed by Western blotting (Figure 5A). The results showed that, compared with the LPS group, the expression of P-JAK2 and P-STAT3 were decreased in the BCTS-CM group. Furthermore, the addition of IL-6 to the BCTS-CM group increased the expression of P-JAK2 (*P* = 0.0187) and P-STAT3 (*P* = 0.0215), while treatment with the JAK2-specific inhibitor AG490 further reduced their expression (P-JAK2, *P* = 0.012; P-STAT3, *P* = 0.0353) (Figures 5B,C). These findings suggest that BCTS-CM attenuates the activation of the IL-6/JAK2/STAT3 signaling pathway, thereby exerting neuroprotective effects.

### 3.6 BCTS-CM treated with BCTS protects PC12 cells from LPS-induced damage and oxidative stress

**FIGURE 6:**
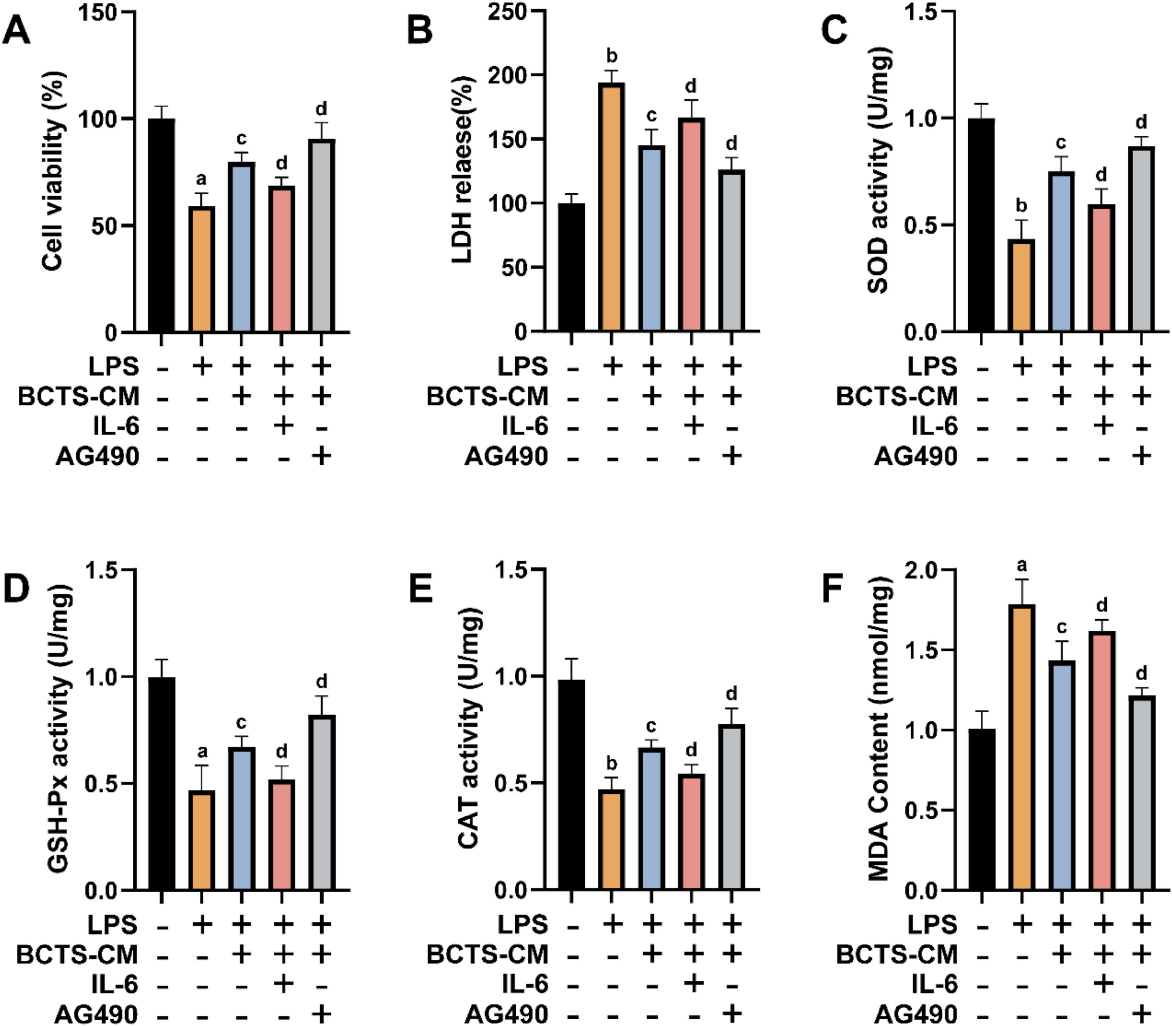
BCTS inhibited LPS-induced damage and oxidative stress in PC12 cells. **(A)** Statistical analysis of PC12 cell viability. **(B)** Statistical analysis of LDH release in PC12 cells. **(C–F)** Statistical analysis of SOD activity, CAT activity, GSH-Px activity, and MDA content in PC12 cells. Data are expressed as mean ± SEM, *n* = 5 vs. Con group, ^a^*P* < 0.05,^b^*P* < 0.01 vs. LPS group, ^c^*P* < 0.05 vs. BCTS-CM group, ^d^*P* < 0.05.

The protective effects of BCTS-CM on PC12 cells subjected to LPS-induced damage and oxidative stress were evaluated. Compared to the Con group, LPS treatment significantly decreased PC12 cell viability. In contrast BCTS-CM group exhibited a higher cell viability than the LPS group. Notably, cell viability in the BCTS-CM + IL-6 group was lower than in the BCTS-CM group (*P* = 0.0183), whereas the BCTS-CM + AG490 group showed a significant increase in viability compared to the BCTS-CM group (*P* = 0.0197) (Figure 6A). The measurements of LDH release, an indicator of cellular damage, displayed an opposite trend to the cell viability, with the LPS group showing the highest LDH release, indicating significant cell damage, while BCTS-CM treatment effectively reduced LDH release. However, the addition of IL-6 to the BCTS-CM group led to increased LDH release (*P* = 0.0164), while AG490 treatment resulted in a further reduction compared to the BCTS-CM group alone (*P* = 0.0398) (Figure 6B). The activity of antioxidant enzymes SOD, CAT, and GSH-Px, which are crucial for combating oxidative stress, were also assessed. BCTS-CM treatment restored their enzyme activity, which had been diminished in the LPS group. In contrast IL-6 addition to the BCTS-CM group decreased enzyme activity (SOD, *P* = 0.0303; GSH-PX, *P* = 0.302; CAT, *P* = 0.223), an effect that was reversed by AG490 treatment in the BCTS-CM group (SOD, *P* = 0.0437; GSH-PX, *P* = 0.314; CAT, *P* = 0.461) (Figures 6C–E) Conversely, MDA levels, a marker of lipid peroxidation and oxidative stress, displayed an opposite trend, with MDA content significantly lower in the BCTS-CM group compared to the LPS group but increasing with the addition of IL-6 (*P* = 0.0417), while AG490 treatment in the BCTS-CM group reduced MDA levels (*P* = 0.0132) (Figure 6F). These findings suggest that BCTS-CM treated with BCTS confers protection to PC12 cells against LPS-induced damage and oxidative stress, potentially through modulation of the IL-6/JAK2/STAT3 signaling pathway.

### 3.7 BCTS alleviates apoptosis in PC12 cells by inhibiting the IL-6/JAK2/STAT3 signaling pathway

**FIGURE 7:**
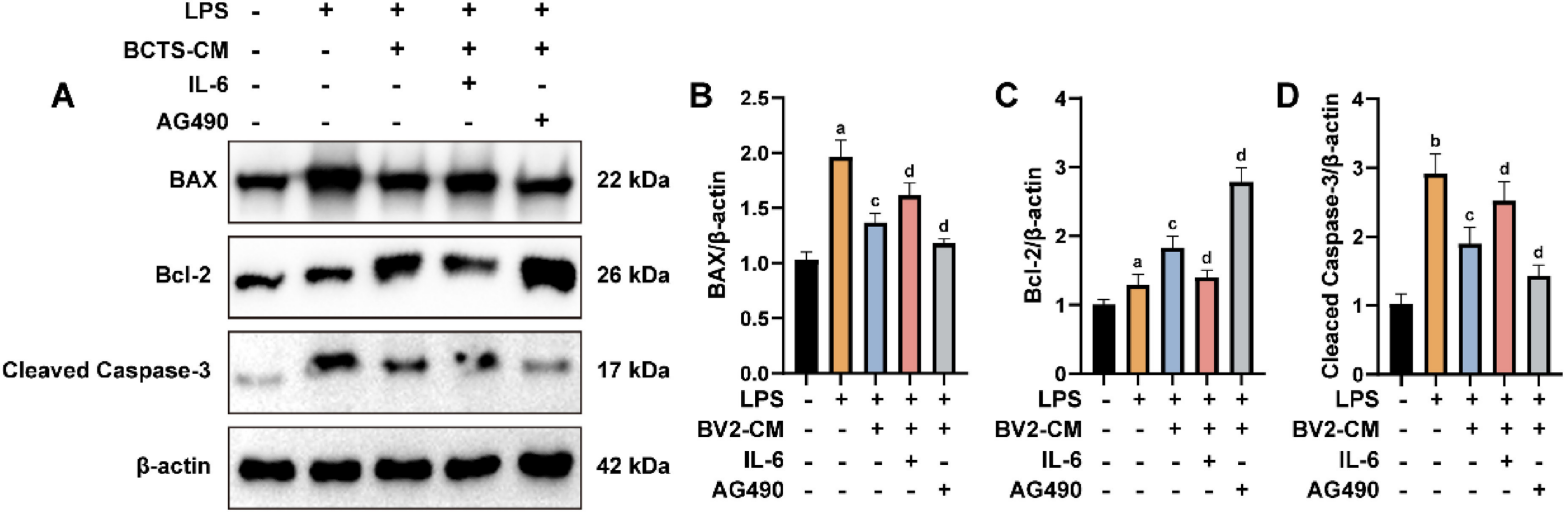
BCTS reduced apoptosis in PC12 cells. **(A)** Western blot results BAX, Bcl-2 and Cleaved Caspase-3 expression. **(B–D)** Statistical analysis of BAX, Bcl-2 and Cleaved Caspase-3. Data are expressed as mean ± SEM, *n* = 5 vs. Con group, ^a^*P* < 0.05,^b^*P* < 0.01 vs. LPS group, ^c^*P* < 0.05 vs. BCTS-CM group, ^d^*P* < 0.05.

To investigate the role of the IL-6/JAK2/STAT3 signaling pathway in PC12 cell apoptosis, the expression of apoptosis-related proteins, including BAX, Bcl-2, and Cleaved Caspase-3, were detected by Western blot (Figure 7A). The results showed (Figures 7B–D) that compared with the LPS group, the expression of BAX and Cleaved Caspase-3 were decreased, whereas Bcl-2 expression was increased in the BCTS-CM group. However, supplementation with IL-6 in the BCTS-CM group reversed these effects, leading to elevated BAX (*P* = 0.0407) and Cleaved Caspase-3 (*P* = 0.0207) levels and decreased Bcl-2 expression (*P* = 0.0103). In addition, treatment with AG490 produced opposite effects, further decreasing BAX (*P* = 0.0469) and Cleaved Caspase-3 (*P* = 0.0318) expression and increasing Bcl-2 expression (*P* = 0.0115). These results suggest that BCTS may alleviate apoptosis in PC12 cells by inhibiting the IL-6/JAK2/STAT3 signaling pathway.

### 3.8 BCTS provides neuroprotection to PC12 cells through the inhibition of the IL-6/JAK2/STAT3 signaling pathway

**FIGURE 8:**
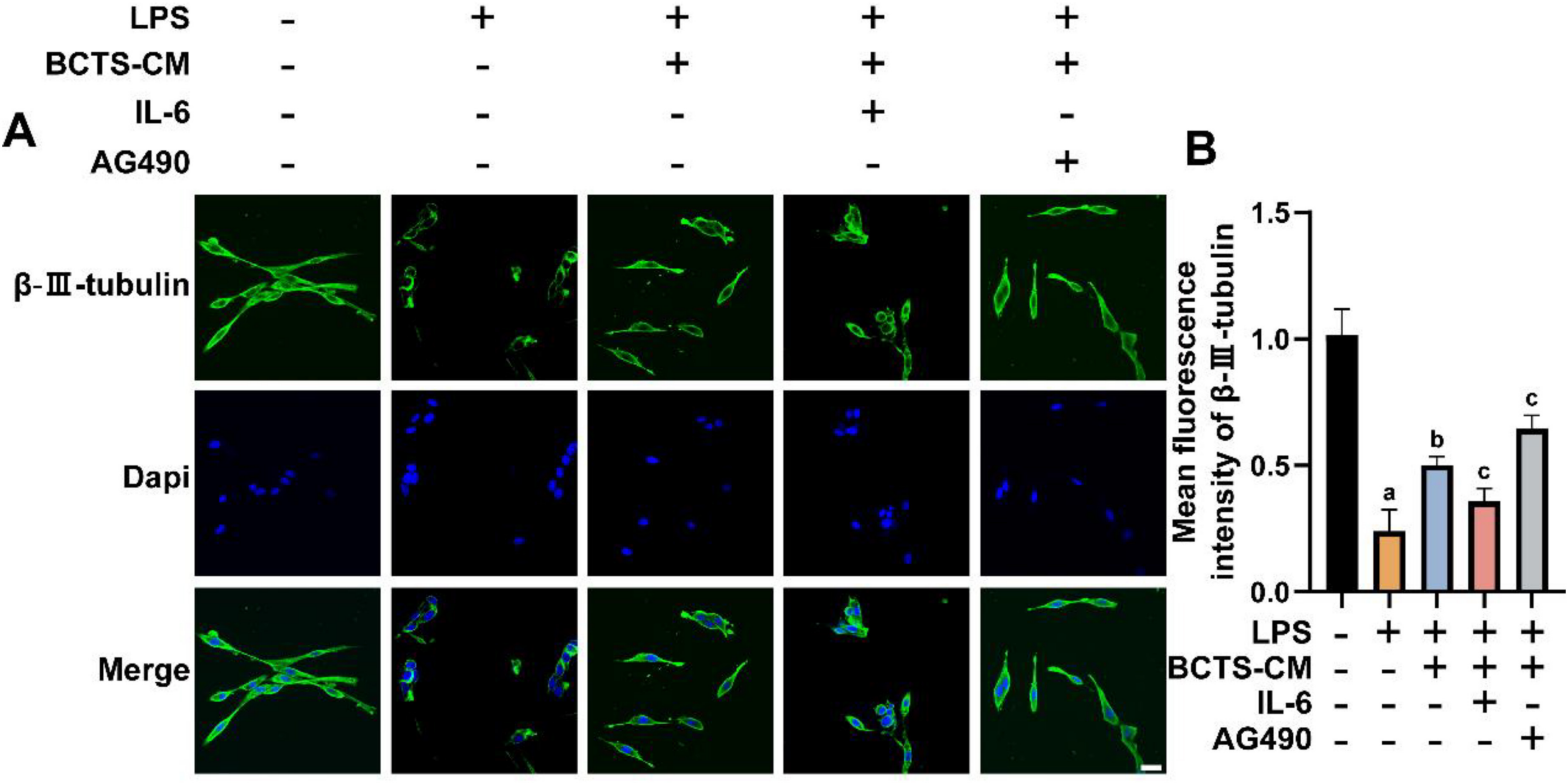
BCTS exerted neuroprotective effects by inhibiting the IL-6/JAK2/STAT3 signaling pathway. **(A)** Immunofluorescence images of β-III-tubulin expression in PC12 cells. **(B)** Statistical analysis of β-III-tubulin expression. Data are expressed as mean ± SEM, *n* = 5. Scale bar = 20 μm vs. Con group, ^a^*P* < 0.05 vs. LPS group, ^b^*P* < 0.05 vs. BCTS-group, ^c^*P* < 0.05.

To investigate the effects of BCTS-mediated inhibition of the IL-6/JAK2/STAT3 signaling pathway on neurons, β-III-tubulin expression in PC12 cells was assessed by IF (Figures 8A,B). The results revealed that the fluorescence intensity of β-III-tubulin was lowest in the LPS group, indicating severe neuronal damage. In comparison, the fluorescence intensity of β-III-tubulin in the BCTS-CM group was higher than in the LPS group but lower than in the Con group. Additionally, the β-III-tubulin fluorescence intensity in the BCTS-CM + IL-6 group was lower than in the BCTS-CM group (*P* = 0.003), while the intensity in the BCTS-CM + AG490 group was higher than in the BCTS-CM group (*P* = 0.0312). These findings suggest that BCTS, by inhibiting the IL-6/JAK2/STAT3 signaling pathway, can enhance β-III-tubulin expression and promote neuronal repair.

### 3.9 BCTS reduces microglial activation and attenuates the inflammatory response at the site of spinal cord injury

**FIGURE 9:**
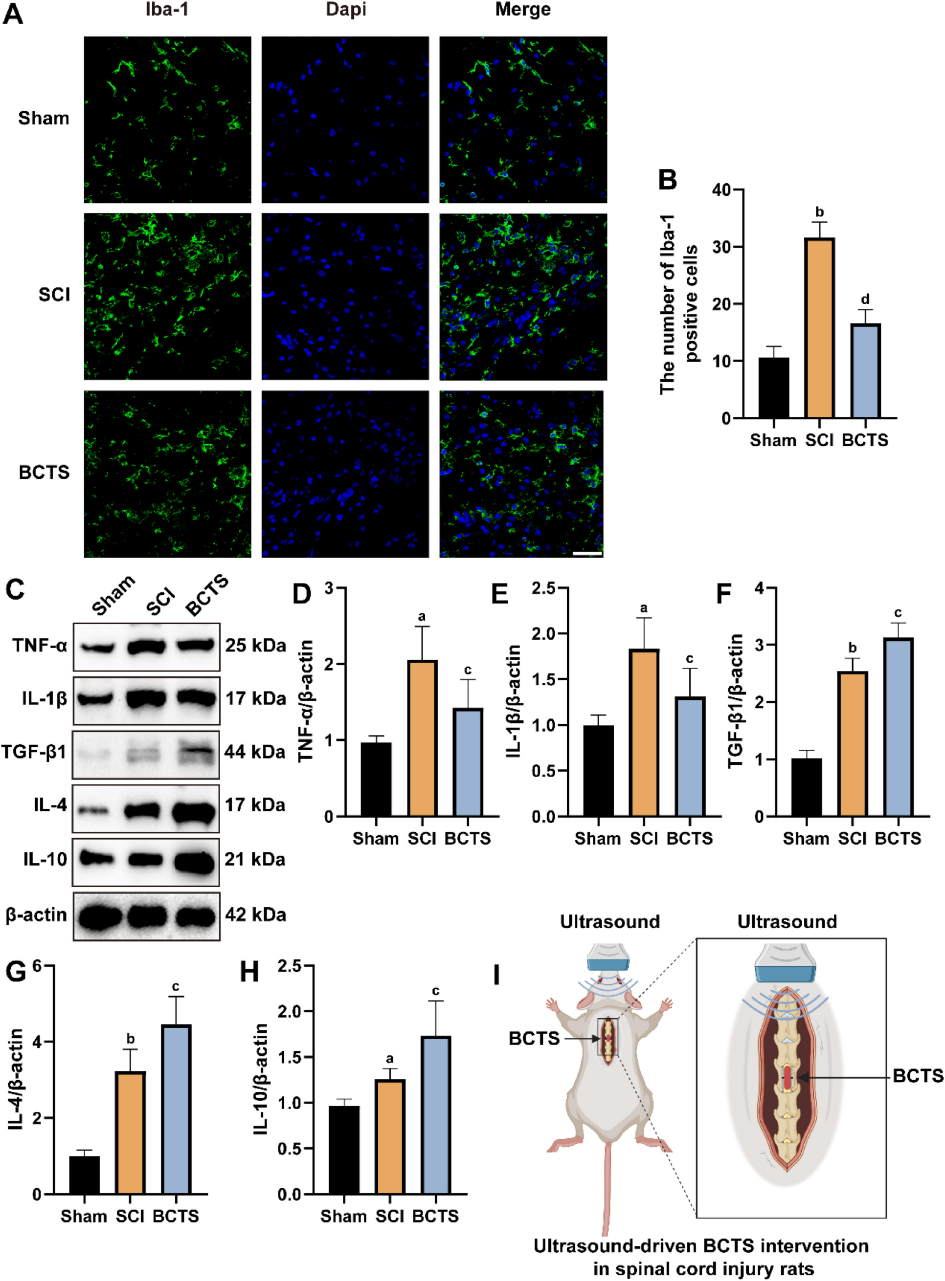
BCTS attenuates microglial activation and inflammation after SCI. **(A,B)** Immunofluorescence images and Statistical analysis of Iba-1 in SCI site. **(C–H)** Western blot results and statistical analysis of TNF-α, IL-1β, TGF-β1, IL-4, and IL-10 expression. **(I)** Ultrasound-driven BCTS was applied to generate a piezoelectric effect for the intervention in rats with spinal cord injury. Scale bar = 20 μm. Data are expressed as mean ± SEM, *n* = 5 vs. Sham group, ^a^*P* < 0.05, ^b^*P* < 0.01 vs. SCI group, ^c^*P* < 0.05,^d^*P* < 0.01.

To investigate the effects of BCTS on microglial activation and the inflammatory response at the site of spinal cord injury, the expression of Iba-1 and inflammation-related cytokines was assessed by immunofluorescence and Western blot analysis. The results demonstrated that the number of Iba-1^+^cells (*P* = 0.0047) (Figures 9A,B) and the expression of TNF-α (*P* = 0.0219) and IL-1β (*P* = 0.0257) (Figures 9C–F) were significantly reduced in the BCTS group compared to the SCI group. In contrast, the expression of anti-inflammatory cytokines TGF-β1 (*P* = 0.0206), IL-4 (*P* = 0.0102), and IL-10 (*P* = 0.019) were markedly increased (Figures 9G,H). The schematic model is presented in Figure 9I. These findings suggest that BCTS suppresses microglial activation and mitigates the inflammatory response at the injury site, thereby contributing to the improvement of the local inflammatory microenvironment following spinal cord injury.

### 3.10 BCTS reduces the expression of the IL-6/JAK2/STAT3 signaling pathway at the site of spinal cord injury

**FIGURE 10:**
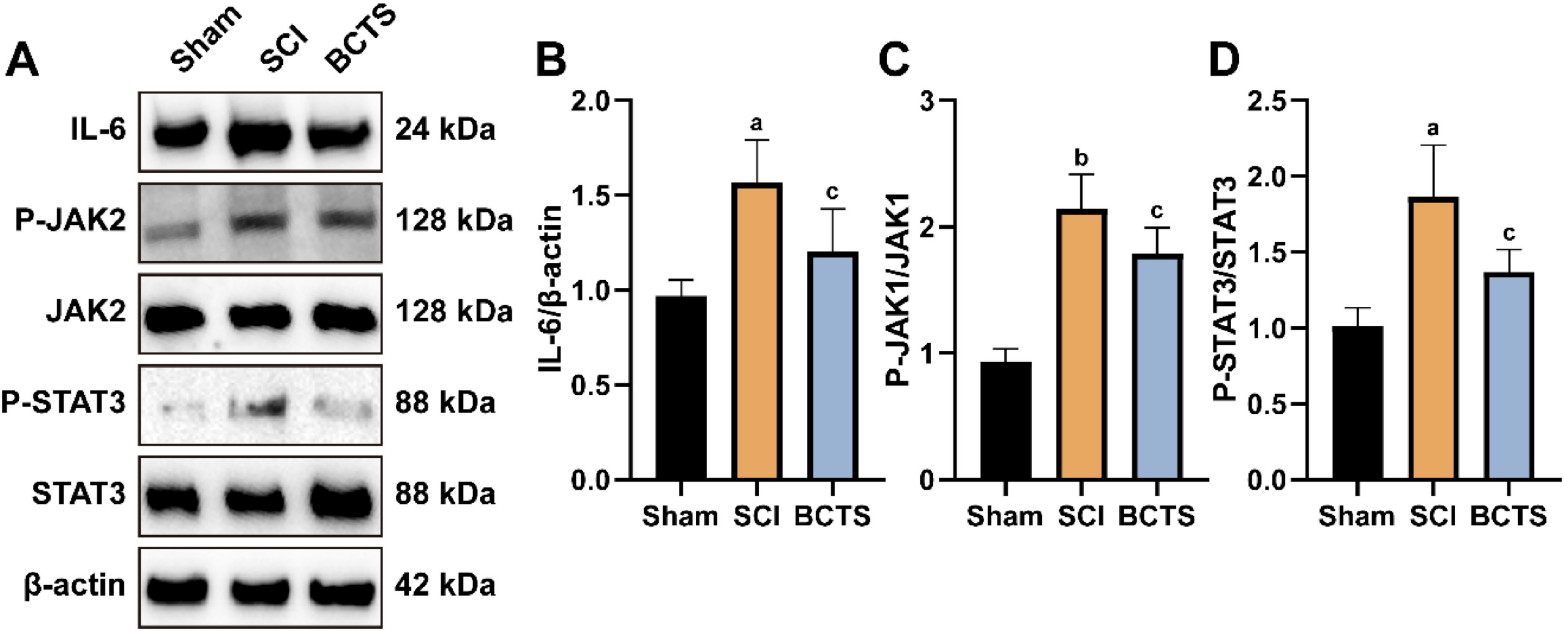
BCTS attenuates the expression of the IL-6/JAK2/STAT3 signaling pathway**. (A)** Western blot results of IL-6/JAK2/STAT3 signaling pathway. **(B–D)** Statistical analysis of IL-6, P-JAK2/JAK2 and P-STAT3/STAT3 expression. Data are expressed as mean ± SEM, *n* = 5 vs. Sham group, ^a^*P* < 0.05, ^b^*P* < 0.01 vs. SCI group, ^c^*P* < 0.05.

To further determine whether BCTS promotes spinal cord repair by modulating the IL-6/JAK2/STAT3 signaling pathway, Western blot analysis was performed to assess the expression of pathway-related proteins in the injured spinal cord. Compared with the SCI group, the BCTS group exhibited reduced expression of IL-6 (*P* = 0.0254), P-JAK2 (*P* = 0.0452), and P-STAT3 (*P* = 0.0122) (Figures 10A–D). These results suggest that BCTS may facilitate spinal cord injury repair by suppressing the activation of the IL-6/JAK2/STAT3 signaling pathway at the lesion site.

### 3.11 BCTS inhibits neuronal apoptosis and preserves spinal motor neurons

**FIGURE 11:**
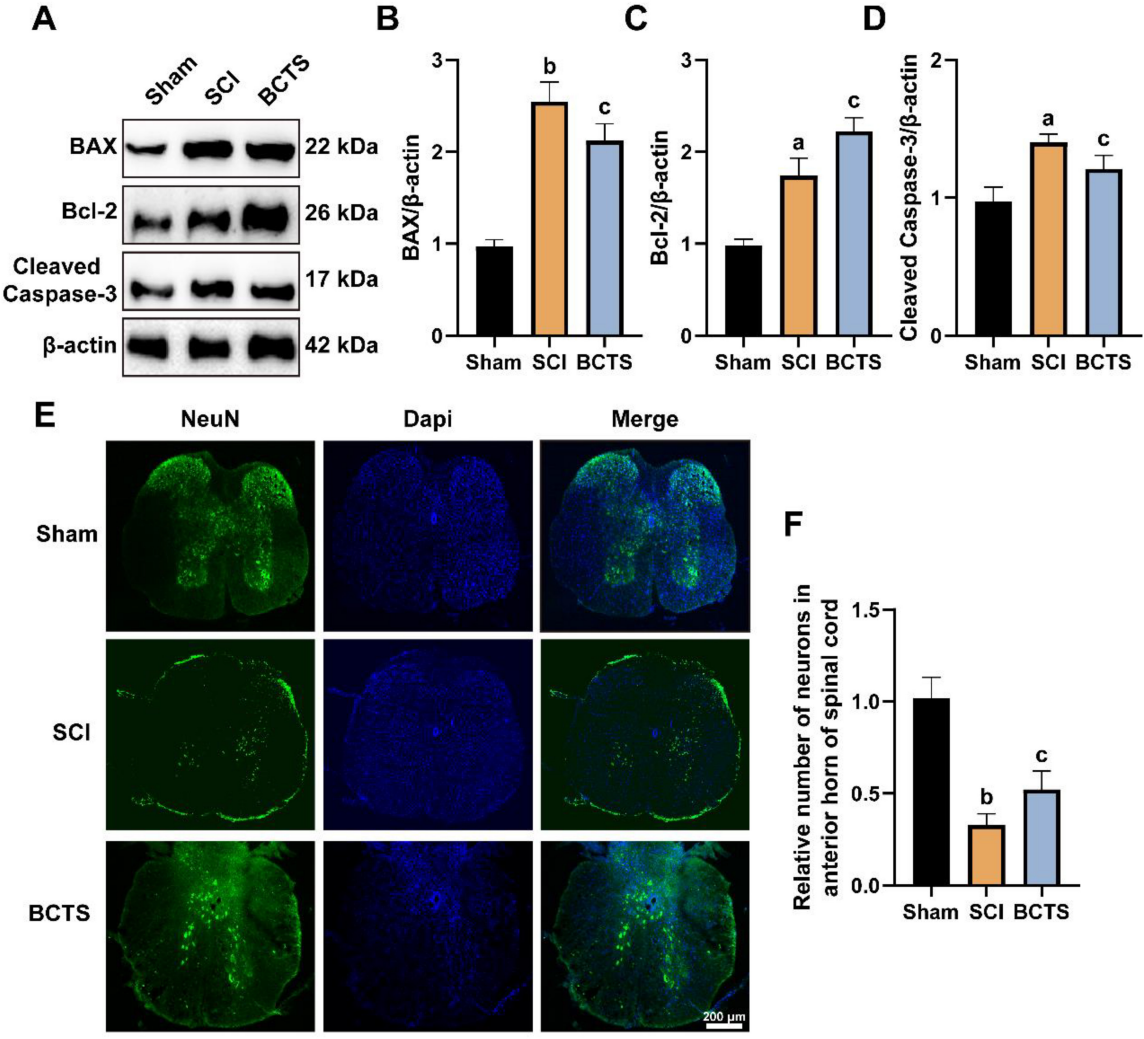
BCTS inhibits neuronal apoptosis and preserves motor function after spinal cord injury. **(A)** Representative Western blot bands showing the expression levels of BAX, Bcl-2, and Cleaved Caspase-3 in the spinal cord tissues of Sham, SCI, and BCTS groups. **(B–D)** Quantitative analysis of the relative optical density for BAX, Bcl-2, and Cleaved Caspase-3. **(E)** Representative immunofluorescence images of the spinal cord anterior horn stained with NeuN (green) and DAPI (blue). **(F)** Quantification of the relative number of surviving neurons in the anterior horn. Data are expressed as mean ± SEM, *n* = 3. Scale bar = 200 μm vs. Sham group, ^a^*P* < 0.05, ^b^*P* < 0.01 vs. SCI group, ^c^*P* < 0.05.

To investigate the molecular mechanism underlying the neuroprotective effects of BCTS, we evaluated the expression of apoptosis-related proteins in the injured spinal cord. Western blot analysis revealed that SCI upregulated the expression of pro-apoptotic proteins BAX and Cleaved Caspase-3, while downregulating the anti-apoptotic protein Bcl-2, indicating the activation of the apoptotic cascade. However, BCTS effectively reversed these trends, decreasing BAX (*P* = 0.0483) and Cleaved Caspase-3 (*P* = 0.0434) levels and increasing Bcl-2 (*P* = 0.0259) expression (Figures 11A–D). Additionally, we performed immunofluorescence staining for NeuN, a marker for mature neurons. In the SCI group, a massive loss of motor neurons was observed in the anterior horn of the spinal cord compared to the Sham group. In contrast, BCTS group attenuated this loss (*P* = 0.0461) (Figures 11E,F).

### 3.12 BCTS promotes motor function recovery after spinal cord injury

**FIGURE 12:**
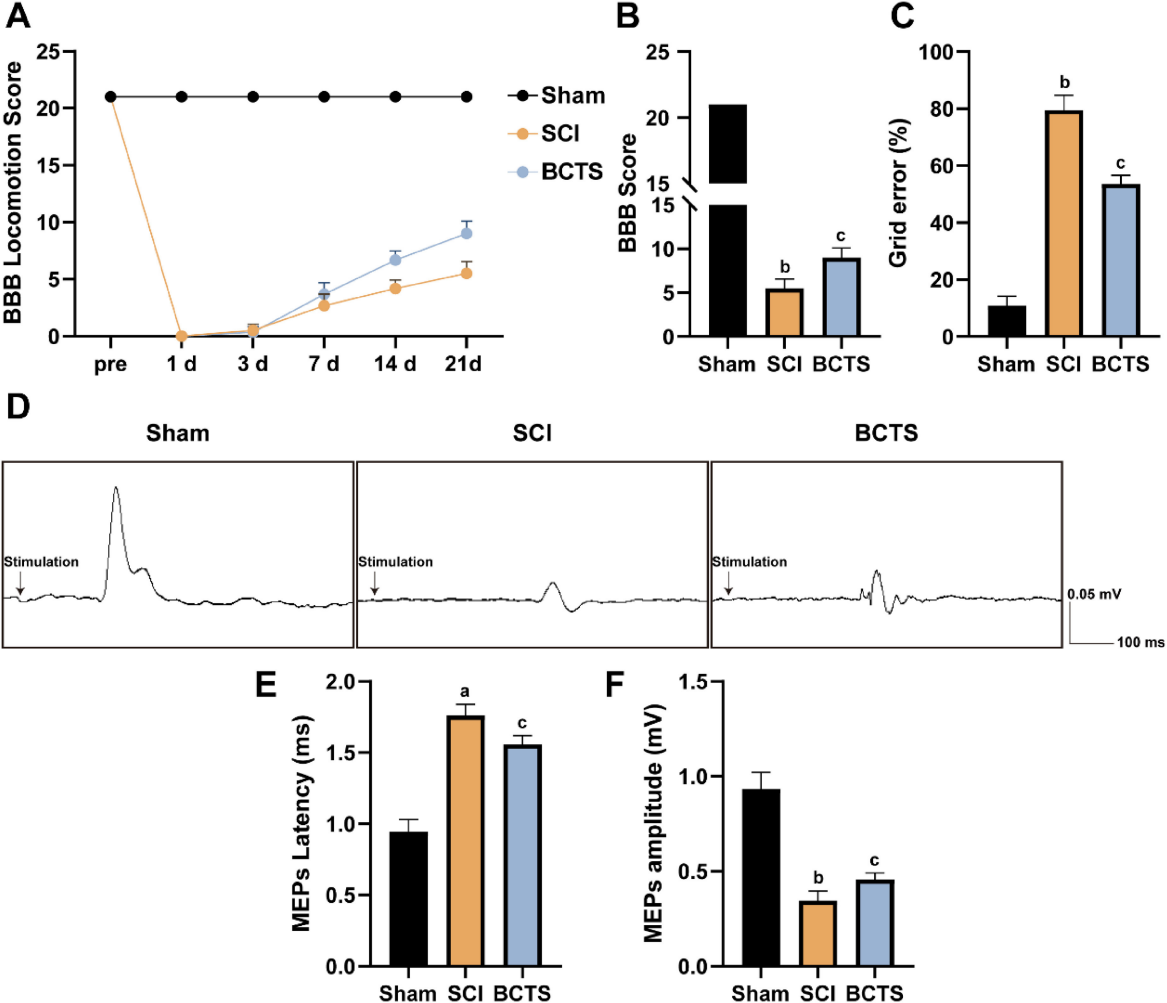
BCTS promotes motor function recovery after spinal cord injury. **(A)** BBB scores at different time points (data are expressed as mean ± SEM, *n* = 6). **(B)** Statistical analysis of BBB score on day 21 post spinal cord injury (data are expressed as mean ± SEM, *n* = 6). **(C)** Statistical analysis of the grid test (data are expressed as mean ± SEM, *n* = 6). **(D)** Representative recordings and analysis of MEPs in different groups at day 21 after SCI. (E,F) Quantification of MEPs latency and amplitude (data are expressed as mean ± SEM, *n* = 6). Scale bar: 0.05 mV/100 ms vs. Sham group, ^a^*P* < 0.01, ^b^*P* < 0.001 vs. SCI group, ^c^*P* < 0.05.

The BBB score results indicated that the BBB score in the BCTS group gradually increased over time following injury and was higher than that in the SCI group (Figure 12A). After 21 days of BCTS intervention, the BBB score was significantly higher in the BCTS group compared to the SCI group (Figure 12B). The grid walking test results (Figure 12C) showed that the SCI group exhibited a significantly higher grid walking error rate, indicating that spinal cord injury severely affected gait control and limb coordination. In contrast, the BCTS group had a lower grid error rate than the SCI group, suggesting that BCTS intervention helps improve gait control and limb coordination following spinal cord injury, thereby partially alleviating the motor dysfunction caused by spinal cord injury. To evaluate electrophysiological changes, motor evoked potentials were recorded in each group (Figure 12D). SCI rats exhibited a markedly prolonged MEP latency and significantly reduced amplitude, indicating impaired corticospinal conduction. After BCTS intervention, the MEP latency was significantly shortened (*P* = 0.0244) and the amplitude markedly increased (*P* = 0.0375) (Figures 12E,F), suggesting improved neural conduction. These findings indicate that BCTS may strengthen axonal excitability and synaptic transmission efficiency within the spinal circuitry.

## 4 Discussion

In this study, we investigated the neuroprotective effects of (Ba,Ca)(Ti,Sn)O_3_-based piezoelectric ceramics, with a particular focus on their influence on microglial polarization and the IL-6/JAK2/STAT3 signaling pathway. Conventional electrical stimulation techniques have been previously shown to alleviate neuroinflammation by reducing microglial toxicity (Goldfarb et al., 2020). These techniques have also proven effective in mitigating knee joint pain and mechanical hyperalgesia in arthritic rats, as well as reducing microglial toxicity (Hahm et al., 2020); however, a significant limitation is their reliance on external power sources, which poses practical challenges for clinical applications. In contrast, BCTS piezoelectric ceramics, with their exceptional piezoelectric properties, overcome the limitations of traditional electrical stimulation methods by generating electrical signals in response to mechanical forces without the need for external power. Crucially, our findings validate the rationale for selecting BCTS over other piezoelectric candidates: unlike polymers (e.g., PVDF) with insufficient output or other lead-free ceramics (e.g., KNN) prone to instability, BCTS provided the robust, stable electromechanical coupling necessary to drive effective immunomodulation within the physiological environment. Additionally, BCTS exhibits excellent biocompatibility and hydrophilicity, which enhances its integration with biological tissues, minimizes irritation and the risk of rejection, and thereby improves both the effectiveness and safety of neuroprotective interventions.

Piezoelectric ceramic materials possess unique electromechanical coupling properties, enabling the conversion of mechanical energy into electrical energy. This characteristic offers distinct advantages in promoting cell proliferation, differentiation, and tissue regeneration. For instance, in bone tissue engineering, piezoelectric ceramics can stimulate bone cell growth and mineralization by mimicking physiological electric fields (Jordan et al., 2020; Yang Y. et al., 2020), while also demonstrating exceptional performance in neural repair by enhancing neuron survival and functional recovery through electrical stimulation (Javidi et al., 2023). In this study, we prepared a piezoelectric ceramic material, BCTS, leveraging electrical stimulation technology. XRD, SEM, and EDX analyses confirmed that the synthesized BCTS exhibits a perovskite phase with a multiphase coexistence, dense grain arrangement, and uniform distribution of doped elements. These findings suggest that the BCTS material exhibits high piezoelectric performance (*d*_33_ up to 450 ± 12 pC/N), thus providing substantial electrical stimulation.

Some reports indicate that US alone exhibits certain anti-inflammatory effects, and that the combination of US with piezoelectric materials produces stronger anti-inflammatory outcomes than US alone (Feltham et al., 2021; Xu et al., 2021). Therefore, to effectively activate the piezoelectric effect of BCTS and enhance therapeutic efficacy, we employed US to drive BCTS, without including a US-only control group. In our LPS-induced BV2 cell damage model, we observed that BCTS effectively inhibits the polarization of LPS-induced microglia toward the pro-inflammatory M1 phenotype, thereby reducing the expression of pro-inflammatory factors. This alignment with existing literature provides a solid experimental basis for the neuroprotective effects of BCTS. Furthermore, as documented in the literature, transitioning microglia to the M2 phenotype in spinal cord injury mouse models promotes the release of anti-inflammatory mediators and recovery of hind limb motor function (Xue et al., 2022), while inhibiting M1 microglia activation and enhancing M2 activation have also been shown to mediate neuroprotection in ischemic stroke (Jin et al., 2023). These insights overcoming the limitations of traditional electrical stimulation methods the rationale for our subsequent investigation into the effects of BV2 on PC12 cells. Additionally, microglial polarization in this study was assessed using a limited set of markers, specifically iNOS and CD86 for M1, and Arg1 and CD206 for M2. While these markers are widely accepted and reasonably reflect M1/M2 polarization (Ji et al., 2018; Tao et al., 2021; Wang et al., 2023), incorporating additional markers such as CD16/32, YM1, and TREM2, or verification via gene expression analyses, would provide a more comprehensive view of microglial heterogeneity and further support the regulatory effects of BCTS on BV2 cell polarization.

Most current research focuses on the potential of specific signaling pathways to regulate microglial polarization, thereby exerting neuroprotective effects. Pathways such as TLR4/NFκB/NLRP3, RhoA/p38MAPK, and BMP6/SMADs have been identified as key modulators of microglial polarization, contributing to neuroprotection (Zhou et al., 2021; Luo et al., 2022; Li et al., 2023; Liao et al., 2023). However, the molecular mechanisms through which microglial polarization mediates neuroprotection remain underexplored. IL-6, a pro-inflammatory cytokine, is known to activate the STAT3 pathway, leading to neuroinflammation and neuronal cell death. In this study, we investigated whether microglia influence neurons via the IL-6/JAK2/STAT3 signaling pathway by treating PC12 cells with BCTS-CM processed with BCTS. Our findings demonstrate that BCTS-CM can alleviate oxidative stress in PC12 cells, reduce LDH release, and enhance β-III-tubulin expression by suppressing the activation of the IL-6/JAK2/STAT3 pathway. Notably, the addition of IL-6 to BCTS-CM yielded opposing outcomes, whereas the addition of AG490 to BCTS-CM treatment mirrored the protective effects of BCTS. These rescue experiments confirm a direct causal link between the BCTS-modulated immune microenvironment and neuronal survival. Additionally, in the spinal cord injury rat model, BCTS exhibited similar effects by reducing microglial activation, decreasing the activation of the IL-6/JAK2/STAT3 signaling pathway, and promoting recovery of motor function. These results suggest that BCTS may exert its neuroprotective effects by reducing IL-6 secretion from microglia, thereby inhibiting the activation of the IL-6/JAK2/STAT3 signaling pathway in neurons. Although the present study demonstrates that BCTS can inhibit the IL-6/STAT3 signaling pathway, focusing solely on IL-6 may oversimplify the underlying mechanism. Previous studies have shown that barium titanate-based piezoelectric materials can reprogram macrophages via the PI3K-Akt-mTORC1 axis and activate BMSC proliferation and osteogenesis through ROS-mediated Wnt/β-catenin signaling (Dong et al., 2025); injectable piezoelectric PVA/CNF/BTO@PDA hydrogels can promote macrophage M2 polarization, suppress pro-inflammatory cytokine expression, and regulate osteotendinous differentiation under H2O2/IL-1β inflammatory conditions (Li et al., 2025); moreover, piezoelectric nanomembranes have been reported to inhibit M1 polarization, enhance M2 polarization, and reduce tissue inflammation via the IL-17A/NF-κB pathway (Dai et al., 2025). These findings suggest that the effects of BCTS piezoelectric materials may involve multiple immunomodulatory and anti-inflammatory pathways beyond IL-6/STAT3. In future studies, we will further investigate BCTS to validate these putative signaling pathways and elucidate its underlying mechanisms more comprehensively.

This conclusion aligns with existing studies that demonstrate the inhibiting of the IL-6/JAK2/STAT3 signaling pathway reduces brain damage and inflammation in ischemic stroke, decreases neurotoxicity, and promotes neuron survival (Park et al., 2017; Zhu et al., 2021; Wang Y. et al., 2022). Our findings also resonate with recent research indicating that NaF-induced BV2 cell activity leads to PC12 cell apoptosis via the IL-1β/JNK signaling pathway (Zhang et al., 2022). Therefore, we propose the following conclusions regarding the BCTS: i. BCTS promotes microglial polarization toward the anti-inflammatory M2 phenotype. ii. BCTS exerts neuroprotective effects by reducing IL-6 secretion from microglia, thereby inhibiting the activation of the IL-6/JAK2/STAT3 signaling pathway in neurons.

Taken together, these findings highlight the therapeutic potential of BCTS for spinal cord injury and provide a rationale for its further development in clinical settings. In terms of clinical translation, multiple factors must be considered to ensure the efficacy and safety of BCTS in neural injuries. BCTS can be applied to the spinal cord surface via miniaturized implantable scaffolds, films, or biodegradable carriers to achieve localized electrical stimulation, modulating inflammation and neuroprotective signaling through its piezoelectric effect. Compared with conventional external electrical stimulation, this strategy offers advantages such as high locality, sustained activity, and independence from external power sources, potentially enhancing both controllability and safety. Long-term biocompatibility and stability are critical for clinical application. Existing data indicate that BCTS induces minimal inflammatory responses and does not disrupt tissue architecture upon contact with spinal cord tissue. Nonetheless, chronic implantation requires further evaluation of immune reactions, potential neural damage, mechanical durability, and electrical performance to ensure sustained therapeutic efficacy. The clinical translation of BCTS still faces challenges, including minimizing invasiveness during implantation, ensuring device compatibility with human tissue, and accounting for variability in injury site, severity, and patient-specific factors. Future studies integrating materials science, neurobiology, and engineering approaches will be essential to optimize the physical properties, implantation strategies, and safety assessments of BCTS, thereby advancing its development toward clinical applications in neural injuries.

Despite the promising findings of this study, several limitations should be acknowledged. First, *in vitro* experiments were conducted using BV2 and PC12 cell lines. Although these cell lines are widely employed as substitutes for microglia and neurons in the literature, they differ from primary cells, which may affect the physiological relevance of the results. Second, while our data suggest that BCTS may exert neuroprotective effects through modulation of the IL-6/JAK2/STAT3 signaling pathway, direct mechanistic validation, such as gene knockdown experiments, is lacking. Single-cell analyses could further elucidate the underlying molecular mechanisms. Third, the observation period in this study was relatively short, and the long-term biocompatibility, stability, and sustained effects of BCTS implantation on neural function remain to be investigated. In addition, the limited sample size may constrain the statistical power and generalizability of our findings. Future studies should verify the *in vitro* findings using primary cells and establish chronic implantation animal models to systematically assess the durability and safety of BCTS in long-term applications. Additionally, integrating multi-omics or single-cell approaches could provide deeper insights into the molecular mechanisms underlying BCTS-mediated neuroprotection, thereby providing a solid scientific foundation for its clinical translation.

In summary, the characterization of (Ba,Ca)(Ti,Sn)O_3_-based piezoelectric ceramics confirms their high piezoelectric performance and suitability for biological applications. BCTS may exert neuroprotective effects by modulating microglial polarization and inhibiting IL-6/JAK2/STAT3 signaling pathways. Our findings underscore the potential of BCTS in neuroprotective therapies. With its unique material properties and biological effects, BCTS holds significant promise for application in neurotherapy.

## Data Availability

The original contributions presented in this study are included in this article/supplementary material, further inquiries can be directed to the corresponding authors.
